# Gene therapy for spinal muscular atrophy: the Qatari experience

**DOI:** 10.1038/s41434-021-00273-7

**Published:** 2021-07-19

**Authors:** Hossamaldein Gaber Ali, Khalid Ibrahim, Mahmoud Fawzi Elsaid, Reem Babiker Mohamed, Mahmoud I. A. Abeidah, Azhar Othman Al Rawwas, Khaled Elshafey, Hajer Almulla, Karen El-Akouri, Mariam Almulla, Amna Othman, Sara Musa, Fatma Al-Mesaifri, Rehab Ali, Noora Shahbeck, Mariam Al-Mureikhi, Reem Alsulaiman, Saad Alkaabi, Tawfeg Ben-Omran

**Affiliations:** 1 Department of Pharmacy, Hamad Medical Corporation, Doha, Qatar; 2 Department of Pediatrics, Sidra Medicine, Doha, Qatar; 3 Weill Cornell Medical College, Doha, Qatar; 4 Qatar Rehabilitation Institute, Hamad Medical Corporation, Doha, Qatar; 5 Department of Pediatrics, Hamad Medical Corporation, Doha, Qatar; 6 Department of Medical Genetics, Hamad Medical Corporation, Doha, Qatar; 7 Center of Rare Disease, Hamad Medical Corporation, Doha, Qatar

**Keywords:** Gene therapy, Neurological disorders

## Abstract

Spinal muscular atrophy (SMA) is an autosomal recessive neuromuscular disorder characterized by hypotonia, progressive muscle weakness, and wasting. Onasemnogene abeparvovec (Zolgensma^®^) is a novel gene therapy medicine, FDA-approved in May 2019 for the treatment of SMA. This study aimed to describe Qatari experience with onasemnogene abeparvovec by reviewing the clinical outcomes of 9 SMA children (7 SMA type 1 and 2 with SMA type 2) aged 4‒23 months treated between November 2019 and July 2020. Children <2 years with 5q SMA with a bi-allelic mutation in the *SMN1* gene were eligible for gene therapy. Liver function (aspartate aminotransferase [AST], alanine aminotransferase [ALT], and total bilirubin), platelet count, coagulation profile, troponin-I levels, and motor scores (Children’s Hospital of Philadelphia Infant Test of Neuromuscular Disorders [CHOP INTEND]), were regularly monitored following gene therapy. All patients experienced elevated AST or ALT, two experienced high prothrombin time, and one experienced elevated bilirubin; all of these patients were asymptomatic. Furthermore, one event of vomiting after infusion was reported in one patient. Significant improvements in CHOP INTEND scores were observed following therapy. This study describes the short-term outcomes and safety of onasemnogene abeparvovec, which is well tolerated and shows promise for early efficacy.

## Introduction

Spinal muscular atrophy (SMA) is an autosomal recessive neuromuscular disorder and the most common fatal inherited disease of infancy resulting from a genetic mutation in the *SMN1* gene located on chromosome 5q13 [1]. Patients with SMA experience progressive muscle weakness and wasting resulting from loss of motor neurons in the spinal cord anterior horn cells [2]. The incidence of SMA is ~1 in 6000–10,000 live births, with the majority (60%) being SMA type 1 [3]. In the Middle East, incidence of SMA has been reported to range from 10 to 193 per 100,000 births [4–7]. SMA incidence of up to 40-fold higher than the Western world [4] is potentially a result of the increased rate of consanguineous marriages in the region. Consanguinity was reported in 45.5% of SMA patients in Egypt [8]. Globally, carrier frequency has been estimated to range between 1 in 45 and 1 in 100 people [3]. The carrier frequency in the region, however, is thought to be much higher, with 1 in 20 normal individuals unrelated to SMA patients being carriers [9].

Nusinersen (Spinraza^®^), the first drug approved for the treatment of SMA, is an antisense oligonucleotide, which increases the amount of functional SMN protein in the central nervous system by alternative splicing of the *SMN2* gene [10]. Nusinersen has been shown to improve motor function in SMA type 1 and 2 patients, as well as increase survival in SMA type 1 patients [10]. Onasemnogene abeparvovec (Zolgensma^®^) is a novel gene therapy for treatment of SMA, which uses the adeno-associated virus vector to deliver the functional *SMN1* gene to the motor neurons [11]. Onasemnogene abeparvovec, approved by the US Food and Drug Administration (FDA) in May 2019, has been shown to improve motor function in infants with severe SMA type 1 [12]. Such treatments are able to slow disease progression or prevent disease development if used prior to symptoms development; nevertheless, multidisciplinary management and support is required to treat the complications of the disease [2].

Studies describing the use of onasemnogene abeparvovec for the treatment of SMA are limited: clinical trials have been undertaken in the USA [11–13]. Furthermore, two retrospective cohort studies in the USA and Germany have also been conducted [14, 15]. Whilst these studies have assessed the safety and efficacy of onasemnogene abeparvovec, there is the need for greater evidence worldwide including data from the Middle East where the incidence of SMA is greater.

The aim of this case series paper is to describe the first Qatari experience with the use of onasemnogene abeparvovec in children with SMA. In this paper, an overview of the treatment pathway, screening for the likely adverse events by assessing liver function, platelet count, coagulation profile, and troponin-I levels following treatment; and assessing changes in the motor score using the Children’s Hospital of Philadelphia Infant Test of Neuromuscular Disorders (CHOP INTEND) [16] score is described.

## Methods

### Study design

In this single-center case series, we describe nine consecutive SMA patients who received the gene therapy at Hamad Medical Corporation, the main governmental hospital in Qatar.

### Ethics (institutional review board)

Parents or caregivers gave written informed consent to receive onasemnogene abeparvovec. The study protocol was approved by the Hamad Medical Corporation Research Ethics Committee in accordance with the Helsinki Declaration of 1964, revised in 2013 (Ethical approval number: MRC-04-20-1141).

The cost of treatment was covered through a government scheme for seven patients and a charity organization supported the other two patients. In addition, the gene therapy was significantly supported by the Center of Rare Disease at Hamad Medical Corporation.

### Setting

The nine recruited patients received the gene therapy in the Pediatric Intensive Care Unit and their post-therapy rehabilitation and follow-up at Qatar Rehabilitation Institute, which are facilities at Hamad Medical Corporation, Qatar. Patients received the therapy between November 2019 and July 2020. Patients were followed up for up to 10 months (at the time of preparing this manuscript).

### Participants

Patients were identified as potential candidates and recruited by the SMA treatment team, which included a geneticist, two pediatric neurologists, and a general pediatrician, after reviewing their genetic and neurological status. Patients under the age of 2 years with 5q SMA with a bi-allelic mutation in the *SMN1* gene and a clinical diagnosis of SMA type 1 or 2 were included. SMA patients aged 2 years and older, those on continuous invasive ventilatory support, those with complete paralysis or those with a low CHOP INTEND score (≤20) were excluded.

### Pre-treatment considerations

After patient selection and screening for inclusion, informed parental consent was obtained. The following pre-treatment steps were followed: baseline laboratory testing was performed, including an AAV9 antibody level; baseline liver function tests, including aspartate aminotransferase (AST), alanine aminotransferase (ALT), and total bilirubin; platelet count; coagulation profile (prothrombin time [PT] and activate tissue thromboplastin); and heart function tests (troponin-I). If the patient’s laboratory results were normal, their weight was checked and a medication request was completed based on their weight and laboratory results. The medication was stored in a special container for each patient, containing a personalized bar code and kept at 3–8 °C for a maximum of 14 days before the infusion. One day before the infusion, the clinical status of the patient was assessed for fever or other signs of infection. As per the protocol, all patients received prednisolone 1 mg/kg/day orally 24 h prior to treatment.

### Treatment

Patients received 1.1 × 10^14^ vg/kg onasemnogene abeparvovec via a single-dose intravenous infusion over 60 min, administered by trained nurses and fully supervised by an experienced clinical pharmacist, with the total volume determined according to the patient’s body weight.

### Peri-treatment considerations

Children received their infusion of onasemnogene abeparvovec in an isolation room within the Pediatric Intensive Care Unit. Parents were allowed to be at beside with their child. Continuous monitoring of the patient’s vital signs (heart rate, respiratory rate, blood pressure, and oxygen saturation) was performed by a nurse. The therapy was handled according to pre-set safety criteria by all staff involved. Patients were monitored for at least 12 h after the infusion to assess and manage any post-infusion events.

### Post-treatment considerations

Following treatment, all patients were kept for a minimum of 12 h for close observation (or longer depending on the child’s need) and were discharged on oral prednisolone (1 mg/kg/day) for 30 days. Prednisolone dose was increased above 1 mg/kg/day if necessary and after 30 days, corticosteroid therapy was tapered by halving the dose every 2 weeks, depending on the clinical and laboratory assessment of the liver function, platelets, and troponin tests. Prolonged treatment with prednisolone was undertaken if necessary.

Regular weekly follow-up of liver function (AST/ALT/total bilirubin), platelet count, coagulation profile, and troponin-I levels were undertaken for up to 3 months or until measurements were within the normal range following full weaning off the steroids. Patients also attended monthly appointments in the multidisciplinary neuromuscular clinic and were all enrolled on an intensive pediatric rehabilitation program.

### Analysis

Elevated transaminases (AST/ALT) were considered as greater than 2× the upper limit of normal (ULN AST > 45 IU/l and ALT > 27 IU/l). High bilirubin levels were defined as >21 μmol/l. Elevated troponin-I levels were considered as >20 ng/l. Low blood platelet count (thrombocytopenia) was defined as a count of <150,000/ml. High PT was defined as >12 s. Motor function was assessed using the CHOP-INTEND scores at baseline and following gene therapy. Following an assessment of the distribution of the data, via a Q–Q plot and Shapiro–Wilk test, a paired *t*-test was used to assess the difference between CHOP-INTEND at baseline and 4–9 months following gene therapy.

## Results

### Participants

Eleven cases were considered for inclusion and two were excluded from the study and from gene therapy: one child was just over the age of 2 years and the other was on full invasive ventilation and had a very low baseline motor score (CHOP-INTEND score of 19).

A total of nine cases were included in the analysis: seven SMA type 1 patients and two SMA type 2 patients. Patient demographics are summarized in Table 1.

**Table 1:** Patient demographics of SMA patients.

Case number	SMA type	SMN2 copy number	Gender	Age at diagnosis	Family history	Consanguinity	Prior therapy (number of doses) [age at start]	Ventilation	Age at infusion	Gene dosing weight (kg)	Temperature at infusion (°C)	O_2_ Saturation at infusion (%)	HR at infusion (beat/min)	RR at infusion (breaths/min)	BP at infusion (mm Hg)
1	1	2	Male	3 m	Yes	Yes	Nusinersen (7) [100 d]	SIMV	1 y 11 m	7.7	36.5	100	100	24	100/60
2	1	2	Male	4 d	Yes	Yes	Nusinersen (7) [10 d]	None	1 y 6 m	7.8	36.5	98	126	28	95/49
3	1	2	Male	37 d	Yes	Yes	Nusinersen (7) [41 d]	BiPAP	1 y 5 m	4.5	36.6	99	115	34	95/41
4	1	2	Male	6 m	No	No	Nusinersen (6) [195 d]	None	1 y 7 m	10.2	36.6	100	96	24	95/50
5	1	2	Male	3 m	Yes	Yes	Nusinersen (4) [102 d]	None	1 y 0 m	8.7	36.8	96	130	40	86/50
6	2	3	Female	21 m	No	No	None	None	1 y 11 m	10.1	36	98	108	23	77/57
7	1	2	Male	2 m	Yes	Yes	Nusinersen (3) [96 d]	None	0 y 4 m	6.0	37.3	100	118	36	101/67
8	1	2	Male	2 m	Yes	Yes	Nusinersen (5) [71 d]	None	0 y 8 m	6.9	36.5	100	120	34	92/62
9	2	3	Female	18 m	Yes	Yes	None	None	1 y 9 m	10.5	36.4	100	121	24	94/52

*BiPAP* bilevel positive airway pressure, *BP* blood pressure, *d* days, *m* months, *HR* heart rate, *RR* respiration rate, *SIMV* synchronized intermittent mandatory ventilation, *SMA* spinal muscular atrophy, *y* year.

One patient had high AAV9 antibody levels initially at the age of 4 months; however, repeated testing at the age of 7 months showed normal results, suggesting maternal transmitted antibodies, and the patient received treatment following the return to normal levels at 7 months. Patient temperatures before and after infusion were normal, no increases in body temperature were observed.

All patients followed the protocol for follow-up in regular weekly appointments; none of the patients withdrew from follow-up.

### Post-treatment blood work monitoring

During the follow-up period, troponin-I levels increased above 20 ng/l in four (44.4%) patients (Supplementary Fig. 1). Two patients experienced an increase above 20 ng/l at week 2, whilst the other two patients experienced an increase above 20 ng/l at weeks 3 and 6. In all patients, the increases were transient and resolved within 1 week. All four patients with elevated troponin-I were asymptomatic. Three patients were reviewed by the cardiologist, had electrocardiograms and echocardiograms performed, which were all reported normal and none of these children required any intervention.

During the follow-up period, AST and ALT increased above 2× ULN in all nine patients (Supplementary Figs. 2 and 3). Case 1 reported an AST level of 1 414 U/l and an ALT level of 1 206 U/l at week 1. Whilst most cases with elevated liver enzyme levels returned to below 2× ULN by week 6, two children (cases 4 and 7) had persistently high levels above ULN, with case 4 having levels above ULN up to week 20. A single case (case 6) reported high bilirubin levels (27 μmol/l) during the follow-up period, which returned to within the normal range by week 2 (Supplementary Fig. 4). The patients with raised liver enzymes or bilirubin remained asymptomatic, whilst they were being closely monitored. Two patients with persistently raised liver enzymes (cases 4 and 7) were further investigated via liver ultrasound, creatine kinase, and viral serology. All tests results were unremarkable and enzyme levels returned to within normal range with further adjustment of their prednisolone doses. Liver enzymes were briefly and only mildly raised in the two children who were treatment naive (did not receive nusinersen prior to onasemnogene abeparvovec gene therapy).

During the follow-up period, PT increased above 12 s in two (22.2%) patients (Supplementary Fig. 5). One of these patients (case 1) had a PT of 12.7 s at baseline. PT was not reduced below 12 s in either patient during the follow-up period; however, these children remained asymptomatic and therefore no further investigations were undertaken.

Four cases reported a drop in platelets below 150,000 cells/ml (Supplementary Fig. 6). In all four cases, the platelet count had returned to normal by week 2. One case (case 6) reported severe thrombocytopenia below 50,000 cells/ml at week 1.

These were all transient drops in platelet levels, which resolved within 1 week. No patients had any symptomatic bleeding and no intervention was needed for any of them.

### Prednisolone treatment

Patients required between 1.5- and 10-months’ prednisolone treatment following infusion, with three patients still undergoing prednisolone treatment. Four patients required prednisolone treatment >1 mg/kg/day: case 4 (1.5 mg/kg/day), case 6 (3 mg/kg/day), case 8 (2 mg/kg/day) and case 9 (2 g/kg/day) due to raised transaminases. One patient has continued prednisolone treatment for up to 10 months (case 4). This patient was the only patient to have continued on nusinersen following onasemnogene abeparvovec therapy which was at the request of the parents resulting from the patient’s favorable response to nusinersen. None of the patients had any serious steroid toxicity as a side effect, including those undergoing longer treatment durations.

### CHOP INTEND score

**Fig. 1:**
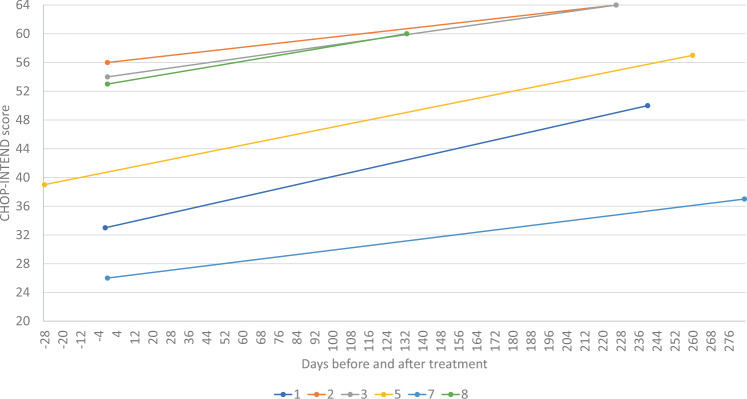
CHOP-INTEND scores Pre and Post gene therapy. CHOP-INTEND scores at baseline and following onasemnogene abeparvovec treatment.

Three patients did not have a CHOP INTEND assessment at baseline as their motor skills at time of gene therapy were above the level of CHOP INTEND, including the two SMA type 2 patients. Of the remaining six patients, CHOP INTEND score increased following onasemnogene abeparvovec gene therapy versus baseline in all patients (mean change 11.8; range 7‒18; paired *t*-test *p* = 0.0015) (Fig. 1).

### Complications and other adverse events

One event of vomiting after infusion was reported in one patient, which lasted for less than 8 h. The patient was observed for more than 12 h and then discharged the following morning. The patient did not receive active intervention and the event was considered an infusion-related event; therefore, there was no need for sepsis work-up.

## Discussion

Whilst the FDA has approved onasemnogene abeparvovec treatment for children under the age of 2 years, clinical trial data was limited to children aged 8 months and younger [14], highlighting the need for data in children up to the age of 2 years. This study has described clinical cases of SMA, aged 4–23 months old, who received onasemnogene abeparvovec gene therapy in a specialized center in Qatar.

All patients experienced elevated liver enzyme levels. Elevated transaminase levels have also been reported in patients from Germany undergoing onasemnogene abeparvovec gene therapy, with one patient experiencing liver damage [15]. Further studies have reported asymptomatic elevation of serum transaminase levels with onasemnogene abeparvovec treatment [11, 13, 14]. Elevated transaminases are thought to be more likely in older or heavier children due to higher total onasemnogene abeparvovec dose received [14, 15]. Whilst there were three older children with higher body weight at gene dosing in our study who required prolonged steroid therapy resulting from raised transaminase levels (cases 4, 6, and 9), a further younger child with a lower body weight also experienced persistently raised liver enzyme levels (case 7). Varying immune response to AAV9 is another potential explanation for raised transaminase levels in some individuals and not others [14]. Liver enzymes have previously been reported to be only briefly and mildly raised in treatment naive children [14], as observed in our study.

There were two cases of high PT, one case of elevated bilirubin, three cases of thrombocytopenia and four cases of elevated troponin-I following gene therapy in our study. Elevations in PT and bilirubin have not been previously observed with onasemnogene abeparvovec gene therapy [11, 13–15]. Thrombocytopenia events have also been reported in previous studies in the USA and Germany; all events were asymptomatic and did not require intervention [14, 15]. Previous reports of elevated troponin-I levels in patients undergoing onasemnogene abeparvovec gene therapy have been described; with troponin-I levels above the normal limit not being linked to any abnormal findings upon further evaluation in these patients [15].

Most patients required between 1.5- and 6-months’ prednisolone treatment following infusion, with the exception of one patient who continued up to 10 months. Prolonged steroid use, up to 6 months, as well as dose increases above 1 mg/kg have been reported in a previous study in Germany resulting from raised transaminase levels or thrombocytopenia [15]. Similarly, prolonged steroid use was observed in over 50% of cases in a USA study, mostly within children older than 8 months of age [14].

CHOP INTEND score improved significantly with therapy in all patients where a baseline assessment was appropriate. Onasemnogene abeparvovec has been shown to improve CHOP INTEND score in a prospective cohort study of SMA type 1 patients by 28.3 points versus a worsening of 15 points for patients not undergoing treatment [13]. This improvement in CHOP INTEND is greater than the improvement seen in our study (mean change 11.8), possibly a result of the lower age at infusion (0.7–7.9 months) compared to our study (4 months to 1 year and 11 months). Furthermore, earlier age at treatment (<3 months) has been shown to enable more rapid CHOP INTEND improvement in Phase I clinical trials [12]. Increased scores are thought to result from rapid production of the SMN protein following treatment [12].

Only one adverse event of vomiting after infusion was reported in this study. Further side effects of onasemnogene abeparvovec gene therapy which have recently been reported, include liver injury or failure [17, 18] and thrombotic microangiopathy [19].

Limitations to the data include missing data as well as varied length of time between gene therapy and motor assessment. Nevertheless, together with previous evidence, this study demonstrates that onasemnogene abeparvovec is well tolerated in SMA patients; however, the number of individuals exposed to onasemnogene abeparvovec gene therapy for SMA is still limited [20]. Barriers associated with such novel therapies might include costs and intricacies of treatment as well as obtaining the necessary expertise [21]. This study demonstrates the importance of government schemes for covering the cost of such treatments. Although newborn screening for SMA is not routinely performed in Qatar, SMA is included in the national premarital genetic screening program and screening is also performed for high-risk families. Such initiatives facilitate earlier diagnosis and treatment of SMA patients. Real-world evidence, including longer follow-up periods, such as the data presented here, and clinical trial data will help generate further safety evidence for onasemnogene abeparvovec treatment in SMA patients.

## Conclusions

This study demonstrates that onasemnogene abeparvovec is well tolerated in SMA patients aged 4–23 months in Qatar. Despite reports of elevated troponin-I, liver enzyme levels (AST and ALT), PT and bilirubin, as well as thrombocytopenia have been documented following gene therapy, patients remained asymptomatic and were monitored closely. Although this study and previous research demonstrates that onasemnogene abeparvovec is largely safe, there are rare reports of severe side effects; therefore, caution is needed when using this gene therapy. Further real-world evidence and clinical trial data are needed to further confirm the safety and efficacy of onasemnogene abeparvovec for treatment of SMA.

## Supplementary information

Supplementary Figure Legends

Supplementary Figures
