# Supplementary material for: Gene therapy for spinal muscular atrophy: the Qatari experience
Source: Gene Ther. 2021 Jul 19;28(10-11):676–80. doi: 10.1038/s41434-021-00273-7 (PMC8599021; doi:10.1038/s41434-021-00273-7)
Supplement: Supplementary file 1 — Supplementary Figure Legends [file 41434_2021_273_MOESM1_ESM.docx]

**Supplementary Figure Legends**

**Supplementary Figure 1.** Troponin-I levels following onasemnogene abeparvovec treatment

**Supplementary Figure 2.** Aspartate aminotransferase levels following onasemnogene abeparvovec treatment

**Supplementary Figure 3.** Alanine aminotransferase levels following onasemnogene abeparvovec treatment

**Supplementary Figure 4.** Bilirubin levels following onasemnogene abeparvovec treatment

**Supplementary Figure 5.** PT following onasemnogene abeparvovec treatment

**Supplementary Figure 6.** Platelet levels following onasemnogene abeparvovec treatment
