# Supplementary material for: Gene therapy for spinal muscular atrophy: the Qatari experience
Source: Gene Ther. 2021 Jul 19;28(10-11):676–80. doi: 10.1038/s41434-021-00273-7 (PMC8599021; doi:10.1038/s41434-021-00273-7)
Supplement: Supplementary file 2 — Supplementary Figures [file 41434_2021_273_MOESM2_ESM.pptx]

## Slide 1
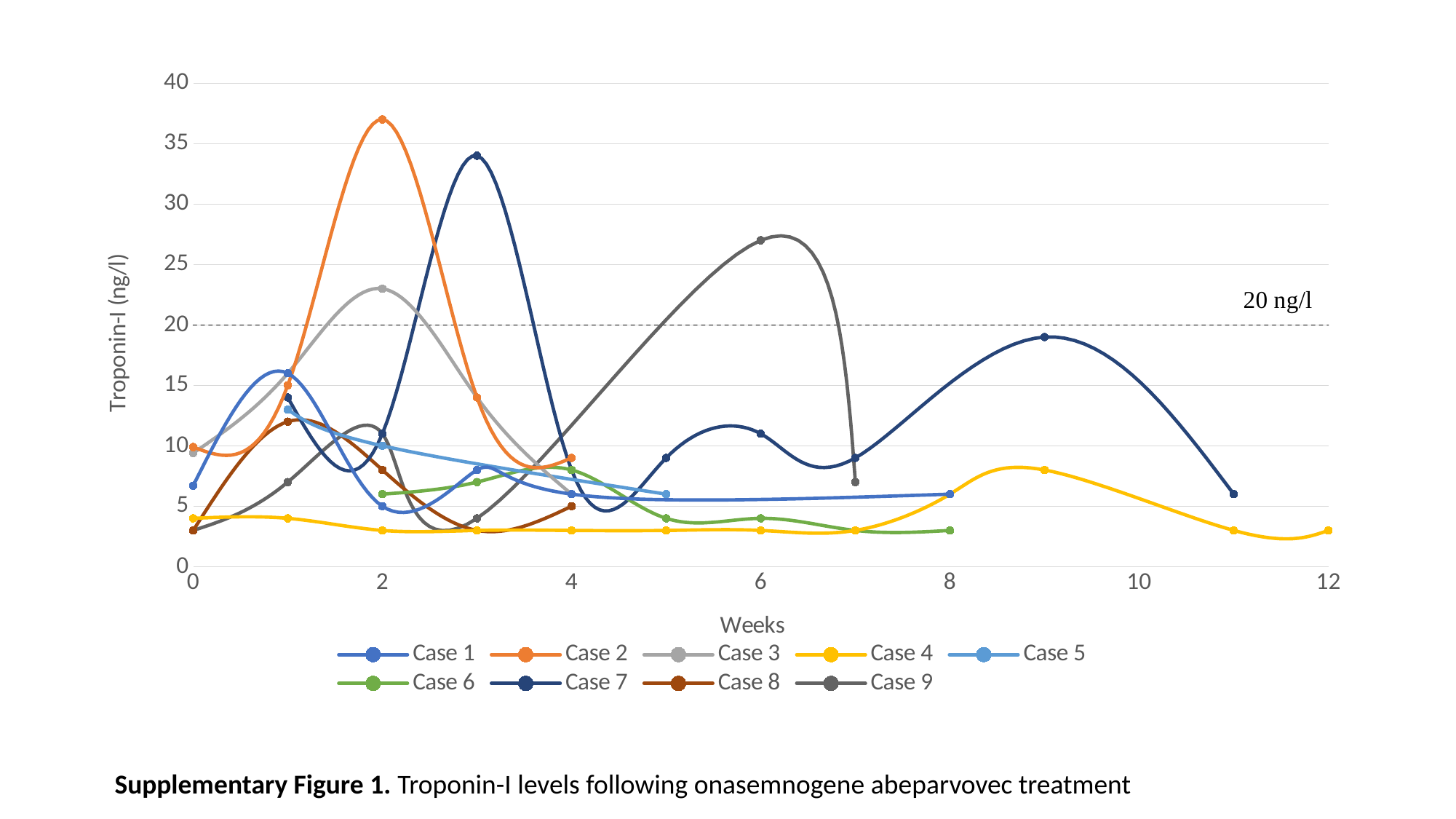

### Chart
| Category | Case 1 | Case 2 | Case 3 | Case 4 | Case 5 | Case 6 | Case 7 | Case 8 | Case 9 | |
|---|---|---|---|---|---|---|---|---|---|---|Supplementary Figure 1. Troponin-I levels following onasemnogene abeparvovec treatment

## Slide 2
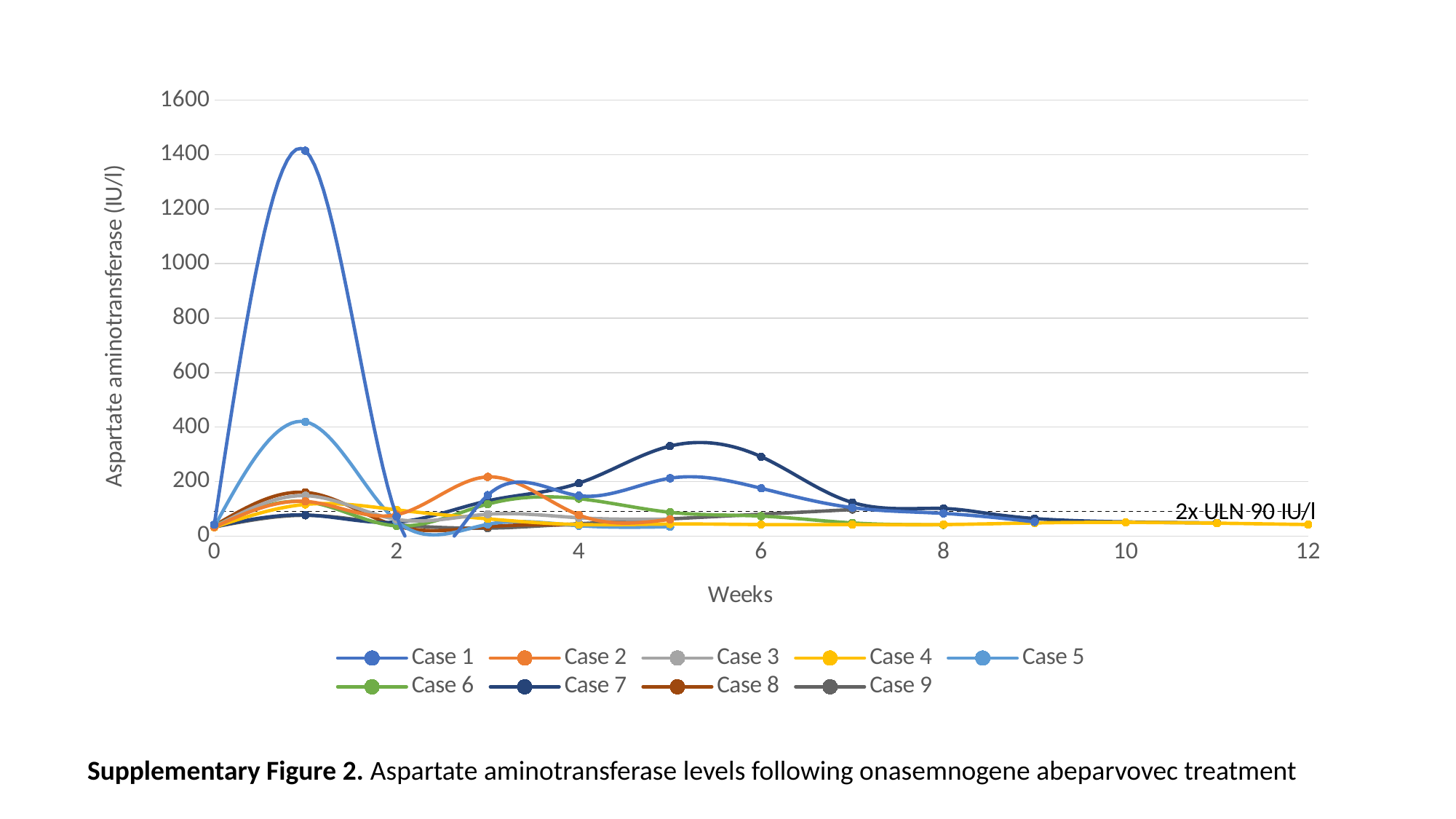

### Chart
| Category | Case 1 | Case 2 | Case 3 | Case 4 | Case 5 | Case 6 | Case 7 | Case 8 | Case 9 | |
|---|---|---|---|---|---|---|---|---|---|---|Supplementary Figure 2. Aspartate aminotransferase levels following onasemnogene abeparvovec treatment

## Slide 3
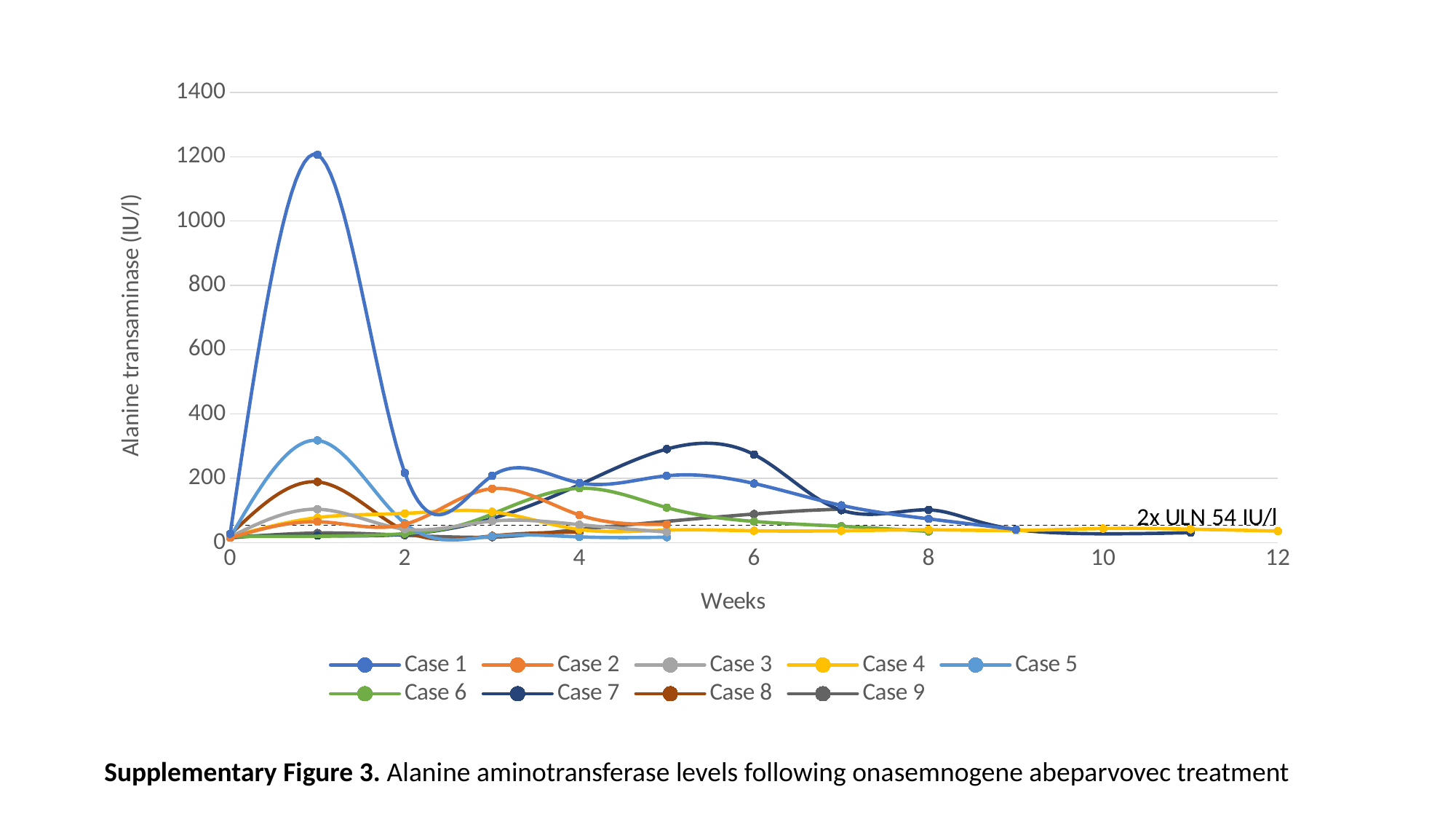

### Chart
| Category | Case 1 | Case 2 | Case 3 | Case 4 | Case 5 | Case 6 | Case 7 | Case 8 | Case 9 | |
|---|---|---|---|---|---|---|---|---|---|---|Supplementary Figure 3. Alanine aminotransferase levels following onasemnogene abeparvovec treatment

## Slide 4
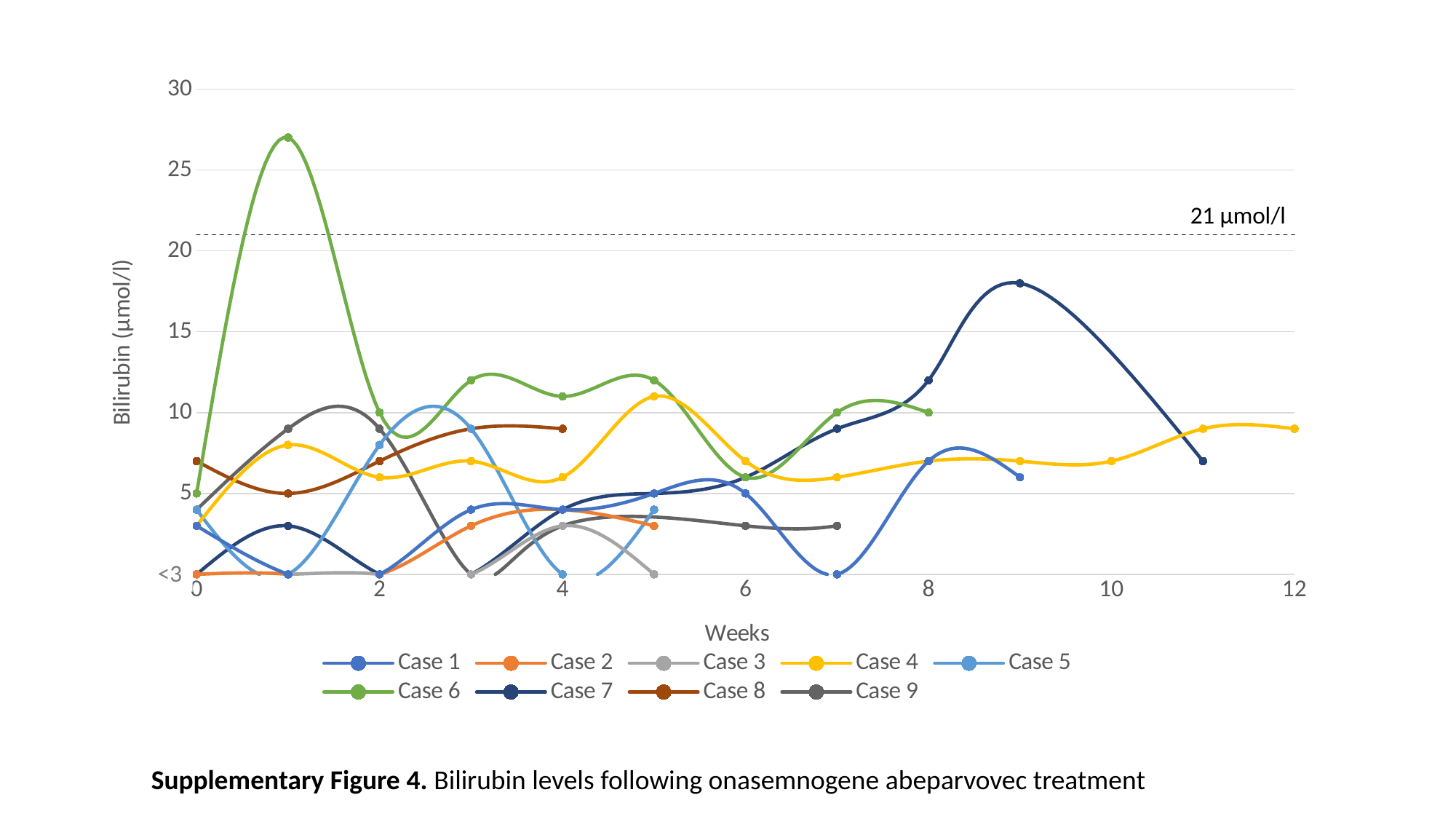

### Chart
| Category | Case 1 | Case 2 | Case 3 | Case 4 | Case 5 | Case 6 | Case 7 | Case 8 | Case 9 | |
|---|---|---|---|---|---|---|---|---|---|---|21 μmol/l
<3
Supplementary Figure 4. Bilirubin levels following onasemnogene abeparvovec treatment

## Slide 5
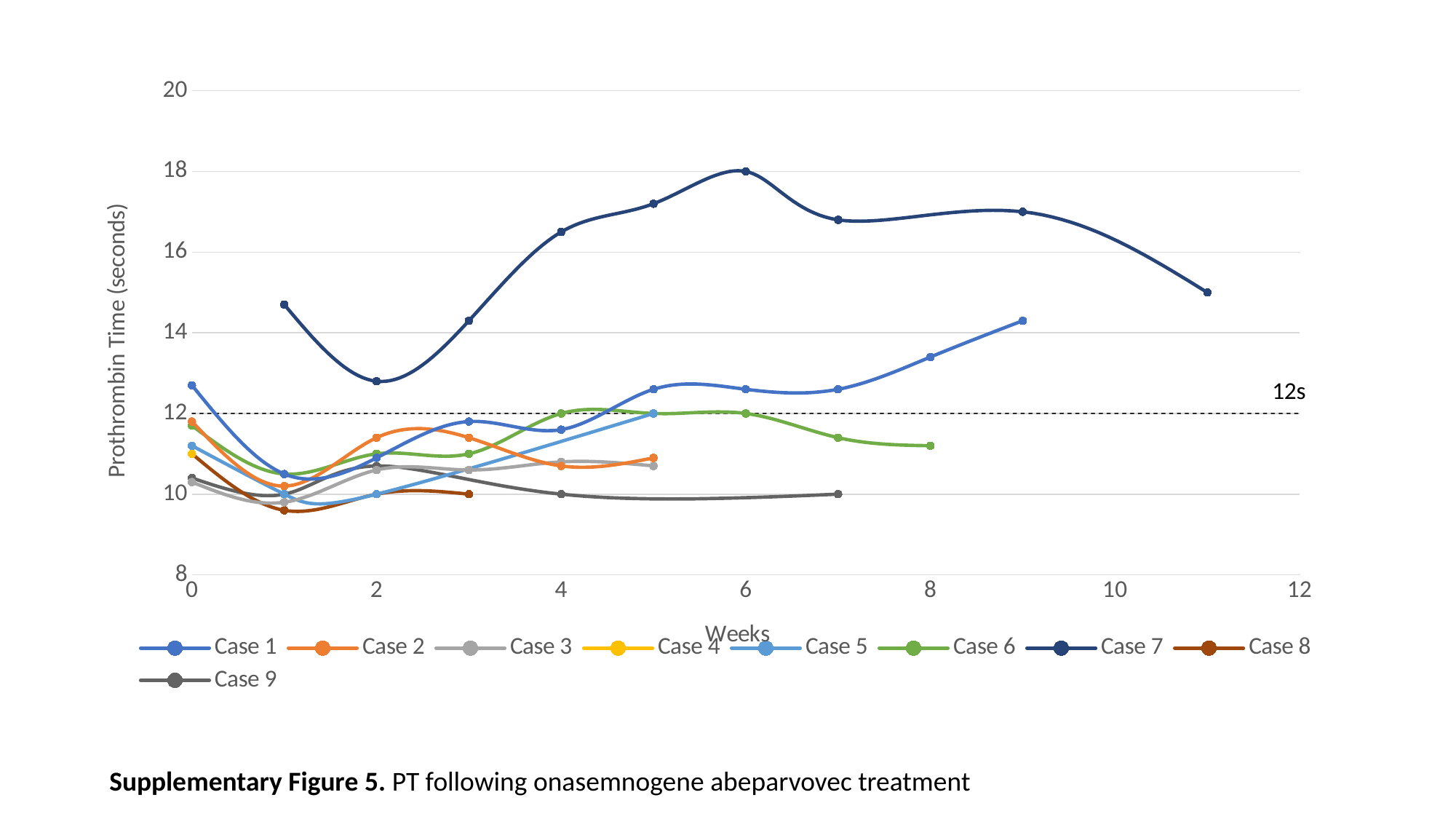

### Chart
| Category | Case 1 | Case 2 | Case 3 | Case 4 | Case 5 | Case 6 | Case 7 | Case 8 | Case 9 | |
|---|---|---|---|---|---|---|---|---|---|---|12s
Supplementary Figure 5. PT following onasemnogene abeparvovec treatment

## Slide 6
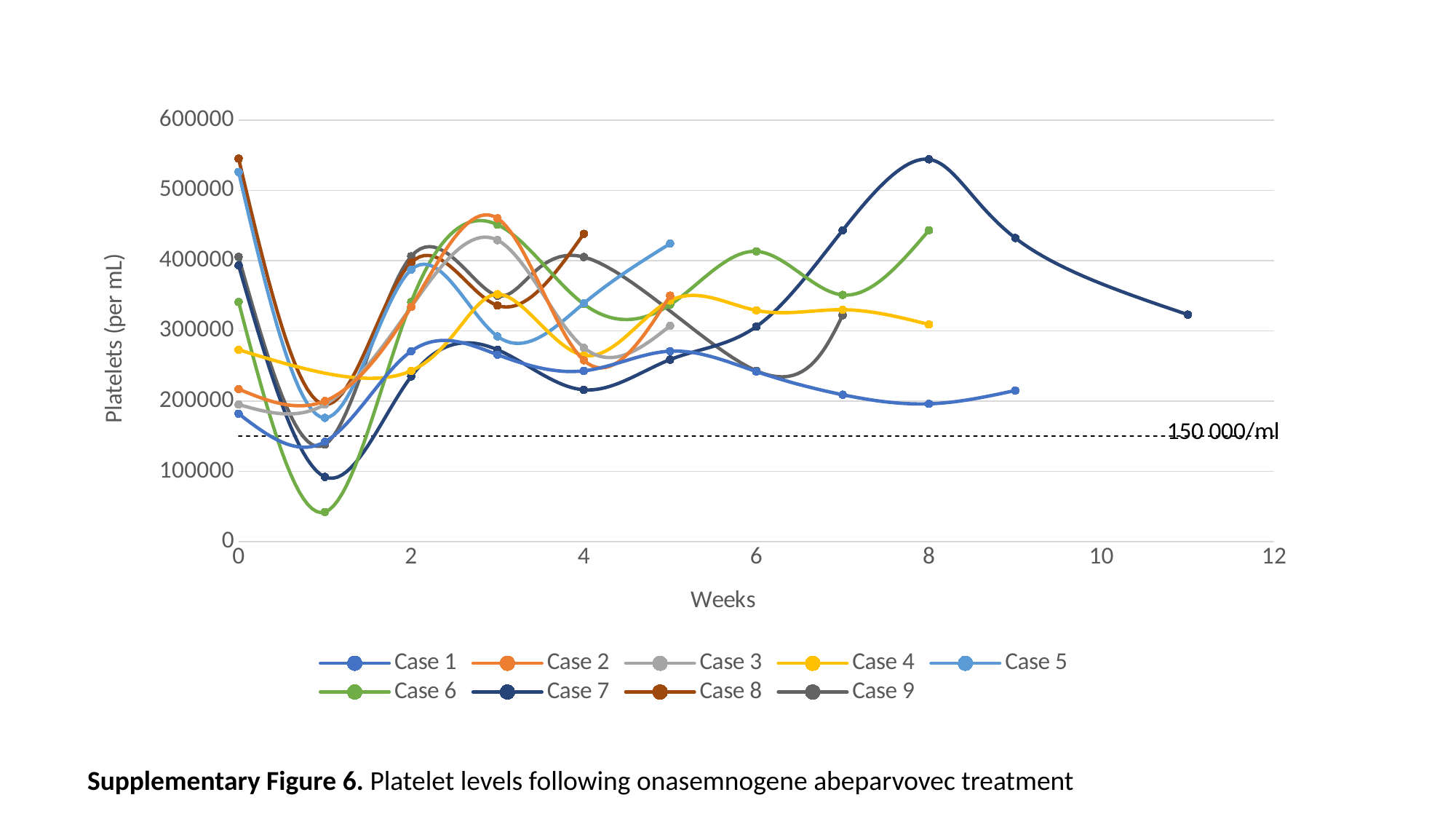

### Chart
| Category | Case 1 | Case 2 | Case 3 | Case 4 | Case 5 | Case 6 | Case 7 | Case 8 | Case 9 | |
|---|---|---|---|---|---|---|---|---|---|---|150 000/ml
Supplementary Figure 6. Platelet levels following onasemnogene abeparvovec treatment
